# Death Associated Protein Kinase (DAPK) -mediated neurodegenerative mechanisms in nematode excitotoxicity

**DOI:** 10.1186/s12868-015-0158-2

**Published:** 2015-04-23

**Authors:** John S Del Rosario, Katherine Genevieve Feldmann, Towfiq Ahmed, Uzair Amjad, BakKeung Ko, JunHyung An, Tauhid Mahmud, Maha Salama, Shirley Mei, Daniel Asemota, Itzhak Mano

**Affiliations:** Department of Physiology, Pharmacology, and Neuroscience, Sophie Davis School of Biomedical Education (SBE), City College of New York (CCNY), The City University of New York (CUNY), New York, NY USA; MS program in Biology, CCNY, CUNY, New York, NY USA; PhD program in Neuroscience, the CUNY Graduate Center, New York, NY USA; Undergraduate program in Biology, CCNY, CUNY, New York, NY USA; Undergraduate program in Biochemistry, CCNY, CUNY, New York, NY USA; Bs/MD program, Sophie Davis SBE, CCNY, CUNY, New York, NY USA

**Keywords:** Ischemia, Glutamate, Excitotoxicity, Neurodegeneration, Death-Associated protein kinase, Autophagy, Peptidyl prolyl isomerase Pin1

## Abstract

**Background:**

Excitotoxicity (the toxic overstimulation of neurons by the excitatory transmitter Glutamate) is a central process in widespread neurodegenerative conditions such as brain ischemia and chronic neurological diseases. Many mechanisms have been suggested to mediate excitotoxicity, but their significance across diverse excitotoxic scenarios remains unclear. Death Associated Protein Kinase (DAPK), a critical molecular switch that controls a range of key signaling and cell death pathways, has been suggested to have an important role in excitotoxicity. However, the molecular mechanism by which DAPK exerts its effect is controversial. A few distinct mechanisms have been suggested by single (sometimes contradicting) studies, and a larger array of potential mechanisms is implicated by the extensive interactome of DAPK.

**Results:**

Here we analyze a well-characterized model of excitotoxicity in the nematode *C. elegans* to show that DAPK is an important mediator of excitotoxic neurodegeneration across a large evolutionary distance. We further show that some proposed mechanisms of DAPK’s action (modulation of synaptic strength, involvement of the DANGER-related protein MAB-21, and autophagy) do not have a major role in nematode excitotoxicity. In contrast, Pin1/PINN-1 (a DAPK interaction-partner and a peptidyl-prolyl isomerase involved in chronic neurodegenerative conditions) suppresses neurodegeneration in our excitotoxicity model.

**Conclusions:**

Our studies highlight the prominence of DAPK and Pin1/PINN-1 as conserved mediators of cell death processes in diverse scenarios of neurodegeneration.

## Background

Excitotoxicity is a neurodegenerative process believed to be the central mediator of brain damage in acute conditions such as brain ischemia and traumatic injury, and an important contributor to a range of chronic neurodegenerative diseases [1-4]. In excitotoxicity, the malfunction of Glutamate (Glu) Transporters (GluTs) [5-7] causes accumulation of Glu in excitatory synapses and exaggerated stimulation of postsynaptic Glu receptors (GluRs) [8]. The excessive influx of ions (especially Ca^2+^) into the postsynaptic neurons leads to their cell death via a spectrum of mechanisms that range from necrosis (at the core of the ischemic damage) to apoptosis or even recovery (at the penumbra). Despite our familiarity with the first few steps in excitotoxicity, our understanding of the steps following Ca^2+^ influx is very limited. Clinical trials using GluRs antagonists ended with disappointment, and recent data suggests that using antagonists to block GluR functions might be counterproductive, because Glu signaling includes both neurotoxic and pro-survival cascades [9,10]. These complications emphasize the need to illuminate cell-death-specific signaling cascades in excitotoxicity downstream of GluRs. A considerable number of such excitotoxic mechanisms have been suggested, but in many cases the data that supports a given suggestion is limited to specific excitotoxic paradigms.

One suggested mediator of excitotoxicity implicated in multiple experimental setups is the CaM-dependent Death Associated Protein Kinase (DAPK) [11-14]. Originally identified by an unbiased screen for mediators of interferon-induced cell death [15], DAPK was later recognized as a molecular switch that controls the choice between cell death processes such as apoptosis and autophagy [16,17]. Moreover, an elaborate web of biochemical interactions and functional connections has been revealed, placing DAPK in a key position at the center of many critical signaling cascades [11,18]. A considerable body of evidence suggests that DAPK also contributes to cell death in excitotoxicity [19], and its inhibition reduces neuronal loss in models of brain ischemia [20]. Several mechanisms have been suggested to explain DAPK’s involvement in excitotoxicity, but the issue remains controversial. One possible mechanism involves DAPK’s regulation of autophagy [17], a process that modulates some neurodegenerative conditions [21]. Alternatively, an intriguing study suggests the involvement of DANGER, a protein that contains a region of homology to the nematode protein MAB-21 and functions as a regulator of the IP_3_R [22]. Indeed DANGER was found to also interact with and inhibit the activity of DAPK [23]. The most cited suggestion in the field attributes the involvement of DAPK in excitotoxicity to the potentiation of Ca^2+^ currents through NR2B/GluN2B subunit-containing complexes of the NMDA- receptor (NMDA-Rs) family of GluRs [24]. This suggestion fits well with a proposed leading role for extrasynaptic NR2B/GluN2B –containing NMDA-Rs in excitotoxicity [25]. However, recently the proposed unique significance of NR2B/GluN2B –containing extrasynaptic NMDA-Rs in excitotoxicity has been brought into question [26-28]. Moreover, an earlier study suggests that in some mammalian neurons DAPK knockout provides protection from excitotoxicity that is not dependent on NMDA-Rs [29]. Indeed, some cases of excitotoxicity are mediated by another family of GluRs, the Ca^2+^ -Permeable AMPA Receptors (CPARs) [30-32]. These observations suggest that if DAPK is widely involved in excitotoxicity, including in cases where NR2B/GluN2B is not a main determinant of neurodegeneration, it might act through additional mechanisms to exert its effect.

We hypothesize that many signaling cascades might be involved in specific cases of excitotoxicity, depending on the exact scenario being used to induce it. However, the key features that constitute the core of the excitotoxic process might be conserved across differences in cell death scenarios and large evolutionary distances, as is the case in apoptosis [33,34] and autophagy [35,36]. We therefore set out to study the role of DAPK in excitotoxicity in our model of CPAR-mediated neurodegeneration in *C. elegans* [37]*,* where GluR-dependent necrosis of central neurons postsynaptic to Glu connections is triggered by knockout (*ko*) of the GluT gene *glt-3* [38] in a sensitized background (*nuIs5* [39]). Indeed, this model has proven effective in identifying core processes that are conserved between nematode and mammalian excitotoxicity [37,40,41]. DAPK is particularly well conserved in *C. elegans* (in 52% sequence homology, presenting all of DAPK’s functional domains, and in its involvement in a number of signaling cascades [42-44]). The nematode DAPK-1 is widely expressed (including in neurons [42]), allowing us to test its involvement in nematode excitotoxicity and to study its mechanism of action. In this study we establish the central role of DAPK in Glu-triggered neurodegeneration in *C. elegans*, suggesting that its function is conserved across evolution and excitotoxic scenarios. We find little or no support for the views that DAPK’s regulation of excitotoxicity is mediated through the modulation of synaptic strength, MAB-21, or autophagy. Instead, we identify PINN-1, the nematode homolog of the DAPK-interaction-partner and phosphorylation-dependent peptidyl-prolyl isomerase Pin1, as an important factor in nematode excitotoxicity.

## Results

### DAPK-1 has a central role in nematode excitotoxicity

**Figure 1:**
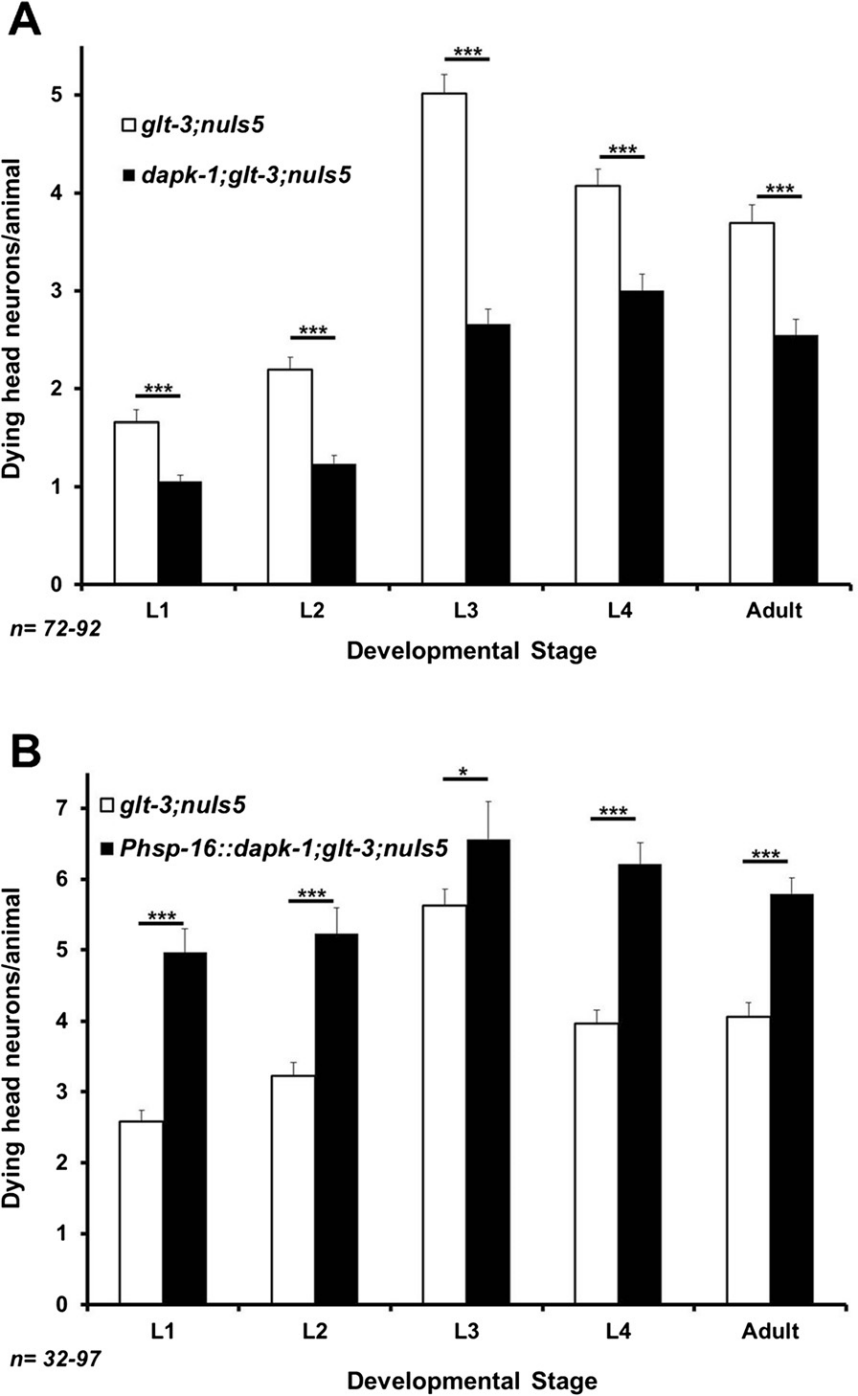
*dapk-1* is an important mediator of nematode excitotoxicity. **A)** Dynamics of neurodegeneration in nematode excitotoxicity during development (using the *glt-3;nuIs5* excitotoxicity model). *dapk-1 ko* mutation suppresses neurodegeneration in all developmental stages, but does not bring it down to background levels. **B)** Overexpression of *wt dapk-1* from a heat-shock promoter enhances neurodegeneration compared to matched controls. In all bar graphs, error bars represent SE. Statistical significance is calculated using z score. *p < 0.05; ***p < 0.01.

To test the involvement of DAPK in nematode excitotoxicity we combined our excitotoxicity strain (*glt-3;nuIs5*) with a deletion allele of the nematode homolog of DAPK, *dapk-1(gk219)*, a gene knockout that was used to confirm the effect of the (ubiquitously expressed) nematode DAPK in autophagy and innate immunity [42,45]. In our previously described model of neurodegeneration we use the sensitizing transgenic modification *nuIs5*, where hyperactive Gαs and GFP are expressed under the *glr-1* promoter in ~30 neurons [39] and cause GluR-independent stochastic degeneration of ~1 of these at-risk neurons per animal. When we add the KO of the GluT gene *glt-3* we observe that more of these at-risk neurons degenerate [37]. The GluT-KO-triggered exacerbated necrosis in *glt-3;nuIs5* is GluR-dependent, and therefore qualifies as nematode excitotoxicity. Nematode excitotoxicity causes neuronal swelling and death that is manifested with characteristic kinetics as gradually and stochastically appearing vacuole-like structures in some of the at-risk postsynaptic neurons. These vacuole-like structures become more abundant during larval development as the Glu signaling system matures (usually reaching up to ~4.5 head neurons/animal at L3), and then decline due to removal of cell corpses by engulfment [37]. We now observe that adding *dapk-1 ko* to this excitotoxicity strain causes a strong and statistically significant suppression of neurodegeneration throughout development (Figure 1A, an additional independent cross gave very similar results, not shown). To further confirm the contribution of *dapk-1* to nematode excitotoxicity, we overexpressed the *wt dapk-1 cDNA* from an extra-chromosomal transgenic construct under a heat-shock promoter [42]. Since heat-shock might affect susceptibility to neurodegeneration, we took special care to compare an exact match of treated animals, without or with the *dapk-1* overexpression transgene. To that end we took advantage of the fact that the random and partial segregation of the non-integrated overexpression construct allows us to compare transgenic and non-transgenic animals on the same plate exposed to the same conditions. We observed that *dapk-1* overexpression resulted in a strong and statistically significant potentiation of necrotic neurodegeneration in postsynaptic neurons in all developmental stages (Figure 1B). Together, our data indicate that DAPK is an important mediator of excitotoxicity in *C. elegans*, suggesting that diverse scenarios of excitotoxicity share a common mechanism that assigns a central role to DAPK.

### *dapk-1* ko does not alter presynaptic release or postsynaptic Glu response

**Figure 2:**
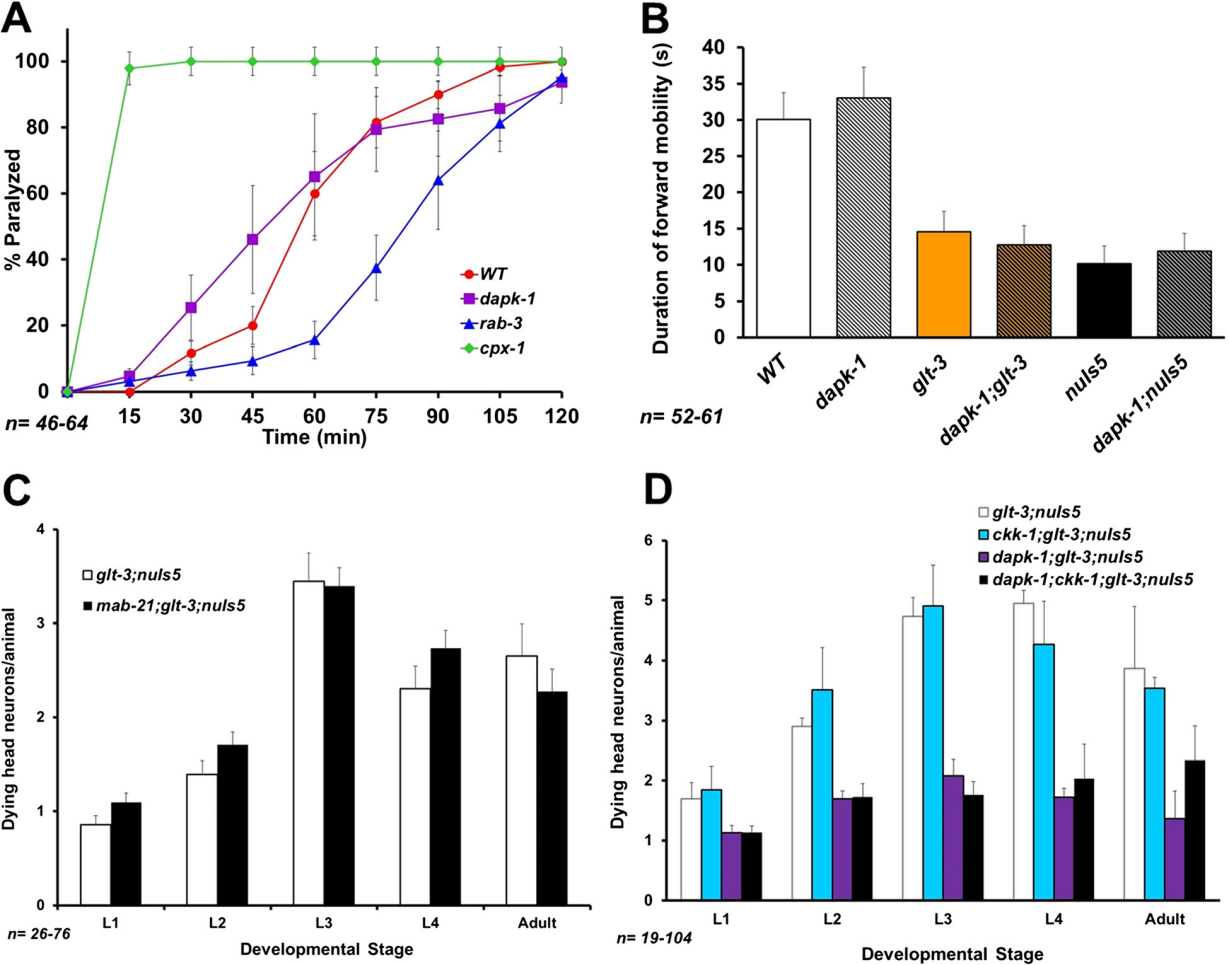
The effect of *dapk-1* does not correlate with several proposed mechanisms. **A)** Aldicarb assay measures the overall activity of the common mechanism of synaptic vesicle release. Complexin (*cpx-1*) mutant is used as example of an oversensitive mutant (where synaptic release is exaggerate), while *rab-3* serves as an example of aldicarb resistant mutant (where synaptic release is abnormally low). *dapk-1* mutants do not show oversensitivity or resistance, suggesting their level of synaptic release is roughly normal. **B)** Duration of forward runs during spontaneous mobility is a sensitive reporter of the overall strength of Glu synapses that control this parameter. Even small changes in the activity or number of GluRs typically modify this behavior. *dapk-1* mutants are not different from normal counterparts in either a WT background, in an excessive Glu stimulation background (*glt-3*), or in the sensitized background (*nuIs5*) used in our excitotoxicity model. These three conditions by themselves do not cause excessive command neuron loss, a loss that might otherwise render the assay uninformative as it is suppressible by *dapk-1*. **C)** A mutation in the gene encoding the DANGER-related protein MAB-21 does not affect nematode excitotoxicity. **D)** A mutation in the gene encoding the nematode homolog of CaMKK does not affect nematode excitotoxicity.

We used two main guidelines in trying to track the mechanism by which *dapk-1* regulates excitotoxic neurodegeneration in the nematode: 1) we looked at previous reports suggesting specific mechanisms for DAPK’s involvement in mammalian excitotoxicity; 2) we inferred from the general map of DAPK’s connectome in other cell processes [18] which additional proteins are plausible candidates for mediating DAPK’s effect in nematode excitotoxicity. One line of evidence suggests that DAPK interacts with, and may regulate the function of, Syntaxin 1A [46]. Syntaxin is part of the general mechanism of vesicular neurotransmitter release, a mechanism that is shared among all neurotransmitters [47]. Therefore, an effect of *dapk-1 ko* on any component of the synaptic vesicle release could lead to DAPK-mediated changes in excitotoxicity levels. However, given the ubiquitous expression of this gene, such an effect of DAPK on the common synaptic vesicle release mechanism will affect the dynamics of neurotransmitter release in all synapses. The study of synaptic vesicle release is very well developed in the nematode, and aldicarb is routinely used in *C. elegans* to identify mutations that cause even modest changes to the general synaptic release mechanism [48]. As aldicarb suppresses the degradation of Acetylcholine in the neuro-muscular junction, it causes animal paralysis with a typical dynamics. Mutations that reduce the activity of the general, common vesicle release mechanism (such as *rab-3* [48]) cause a pronounced resistance to aldicarb (shifting the time-dependent paralysis curve to the right, Figure 2A), while mutations that enhance synaptic release (such as the *ko* of *cpx-1*, which encodes the synaptic vesicle release regulator complexin [49]) cause increased sensitivity to aldicarb (shifting the paralysis curve to the left, Figure 2A). We observed that the sensitivity of *dapk-1* animals to aldicarb is indistinguishable from that of WT animals, suggesting that *dapk-1 ko* does not modify the synaptic release mechanism, as would have been expected of an effect on syntaxin (Figure 2A).

We next asked if DAPK modifies the extent of postsynaptic response in the glutamatergic synapses where excitotoxicity occurs (as reported in mammals, where DAPK was suggested to modify synaptic strength [24]). Two behavioral assays have proven very effective in detecting even small changes in the activity level of glutamatergic synapses in *C. elegans*. These are the Nose Touch (NOT) assay, and the duration of spontaneous forward mobility assay. Therefore, if *dapk-1 ko* causes changes in the specific Glu packaging mechanism in synaptic vesicles (e.g., by affecting vGluTs), or changes the number or activity-level of GluRs in the synapse, this should be reflected in behavioral changes in these assays. In the particular case of *dapk-1*, a secondary phenotype of this mutation, namely overgrowth of cuticle on the animal’s nose [42], makes the nose-touch assay less informative. However, the duration of spontaneous forward mobility depends on the internal balance between forward and backward circuits, and should not be affected by the cuticle aberration. This assay is very reliable in detecting both under-activity and over-activity of the relevant Glu synapses [50-52], allowing us to use it as a proxy measure for Glu synaptic strength. We did not observe any changes in spontaneous mobility triggered by *dapk-1 ko*, either in WT background or in the background of each of the two components (*glt-3* or *nuIs5*) used in our excitotoxicity model to trigger the intensified Glu signaling (Figure 2B). These observations suggest that *dapk-1* has no strong effect on synaptic release or the overall strength of signaling in Glu synapses in *C. elegans*.

### The DANGER-related protein MAB-21 and the CaMKK CKK-1 do not exert strong regulation of nematode excitotoxicity

The characterization of DAPK as a CaM-dependent kinase is particularly intriguing to us, since Ca^2+^ signaling is critical to excitotoxicity in both mammals and nematodes. We therefore examined DAPK-partners that might also be involved in Ca^2+^ signaling. DANGER is a mammalian DAPK inhibitor that regulates Ca^2+^ release from the ER [22,23]. There is no direct, full-length homolog of DANGER in the worm genome, but the core of the mammalian protein is thought to be homologous to the nematode protein MAB-21 [53]. It is therefore reasonable to assume that *mab-21* will be essential to the role of a hypothetic DANGER-like complex in *C. elegans*. However, a mutation in *mab-21* had no effect on nematode excitotoxicity (Figure 2C). Another important protein that binds mammalian DAPK and is involved in Ca^2+^ signaling is CaMKK. However, again, a mutation in the only CaMKK gene in the worm, *ckk-1* [54], had no effect on excitotoxicity (Figure 2D). These results suggest that these two putative Ca^2+^ signaling regulators and DAPK-interaction-partners do not have a large contribution to nematode excitotoxicity.

We also considered the many other proteins that are known to interact with DAPK [18] as possible modulators of excitotoxicity, but we could not assign to many of them high priority because either there was no immediate obvious connection to neurodegeneration (e.g., tropomyosin), there is no clear nematode homolog (e.g., NFκB), the nematode homolog is not known to be active in *C. elegans* neurons (e.g., p53), or the process in which this protein is involved has been shown by us to be not involved in nematode excitotoxicity (e.g., apoptosis [41]). However, DAPK is also known to interact with Beclin1, a key regulator of the evolutionary conserved process of autophagy, suggesting that autophagy-mediated cell death could potentially be an avenue for DAPK to regulate nematode excitotoxicity.

### Autophagy has a minor role in nematode excitotoxicity

**Figure 3:**
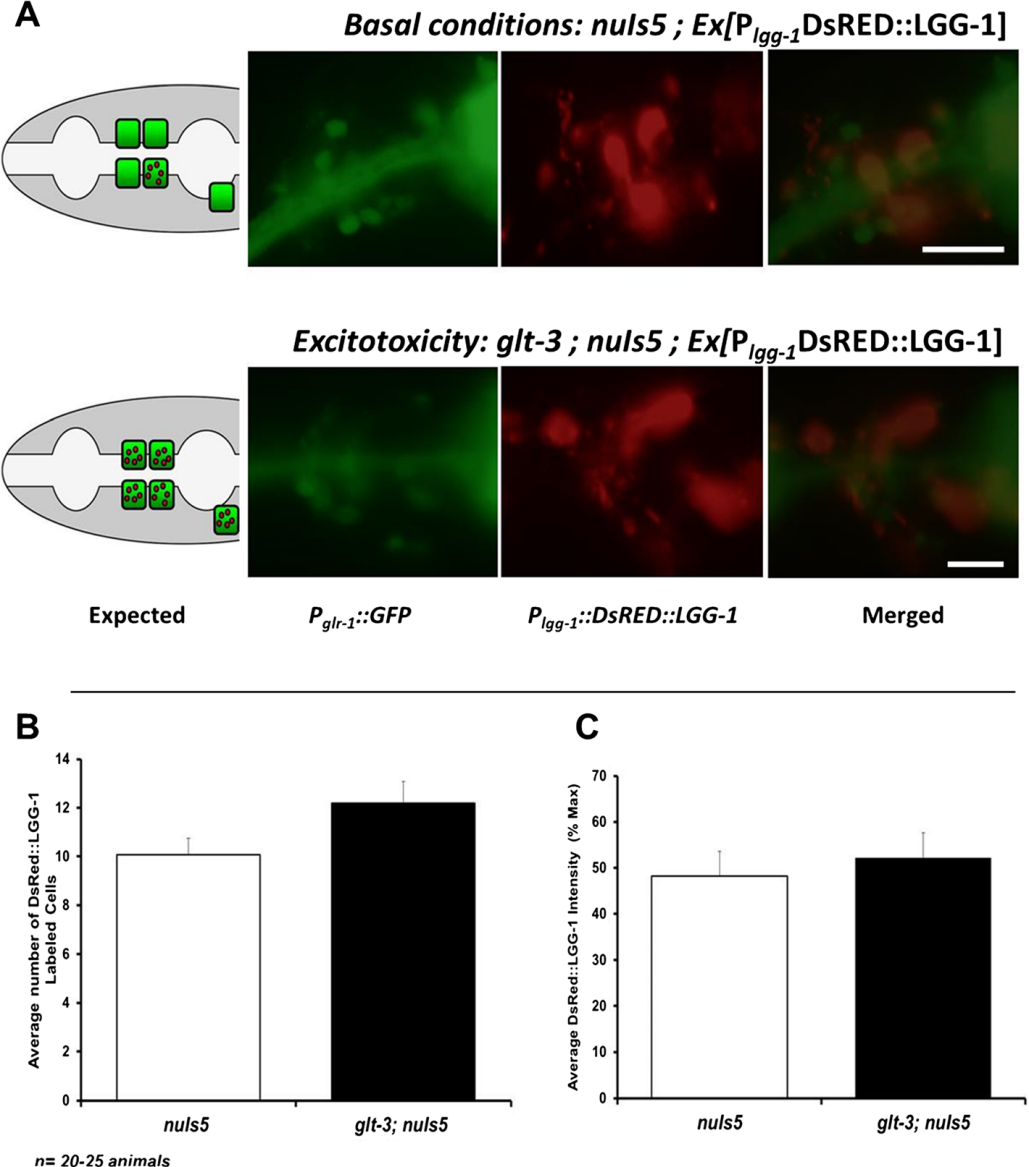
A DsRed::LGG-1 reporter of autophagy does not provide convincing evidence for triggering of autophagy in at-risk neurons exposed to the excitotoxic insult. **A)** Representative images showing at-risk neurons in green (expressing *P*_*glr-1*_ *::GFP*) and DsRed::LGG-1 expressing cells in red. Lateral view, anterior left, dorsal up, illustration on the left describing the results expected from a putative involvement of autophagy in excitotoxicity. Expression of green labeling in the pharynx comes from the co-injection marker for the DsRed::LGG-1 label, expressing *P*_*myo-2*_ *::GFP*. **B)** Analysis of images taken from the two groups shows a similar number of at-risk neurons (green cells) showing DsRed::LGG-1 puncta. The observed small difference is not statistically significant. (*t* test used here) **C)** The average intensity of the DsRed::LGG-1 signal in at-risk neurons (green) in very similar in the two groups.

The interaction between mammalian DAPK and Beclin1 is considered a major avenue for DAPK’s ability to regulate autophagy [11,16,17,55]. Autophagy has been suggested to be an important factor in some neurodegenerative conditions [21,56,57], and has also been shown to be a major contributor to degenerin-mediated neurodegeneration in the nematode [58-60] (where necrotic neurodegeneration is triggered by a constitutively open channel of the DEG/ENaC family [61]). Moreover, *dapk-1* regulates autophagy in *C. elegans* [43]. We therefore set out to determine the role of autophagy in nematode excitotoxicity, using some of the same reagents used to demonstrate autophagy’s role in degenerin-induced neurodegeneration in the worm [58,60,62]. One such reagent is the red fluorescent labeled LC3 –homolog LGG-1 (originally created by the Tavernarakis lab [62], for the current study assigned the strain name IMN21). This reporter indicates elevated autophagy by both the appearance of intracellular puncta and by an overall increase in cellular fluorescence. We crossed IMN21 with our excitotoxicity strain *glt-3;nuIs5*. Since the red fluorescence can appear in any body cell that triggers autophagy, we used the *P*_*glr-1*_*::GFP* marker expressed in our excitotoxicity strains to focus our attention on the postsynaptic neurons that are at risk for neurodegeneration. If autophagy is a strong component of excitotoxicity, we would expect at-risk neurons (labeled with GFP) to express autophagy marker (DsRed) upon exposure to high concentrations of Glu (triggered by the *glt-3 ko*). We counted the number of at-risk neurons that show LGG-1 puncta and measured the overall intensity of DsRed::LGG-1 signal in these neurons. Since autophagy was reported to have a role in the low-level neurodegeneration caused by *nuIs5* alone [60], we concentrated on the added effect of excitotoxicity by comparing DsRed::LGG-1 signals in animals that express only the sensitizing construct *nuIs5* to those of animals in which neurons are subjected to the full excitotoxic insult (*glt-3;nuIs5*). We found that excitotoxicity resulted in only a small and statistically-insignificant increase in LGG-1 puncta, and no change in overall LGG-1 intensity in at-risk neurons (Figure 3A and B).

**Figure 4:**
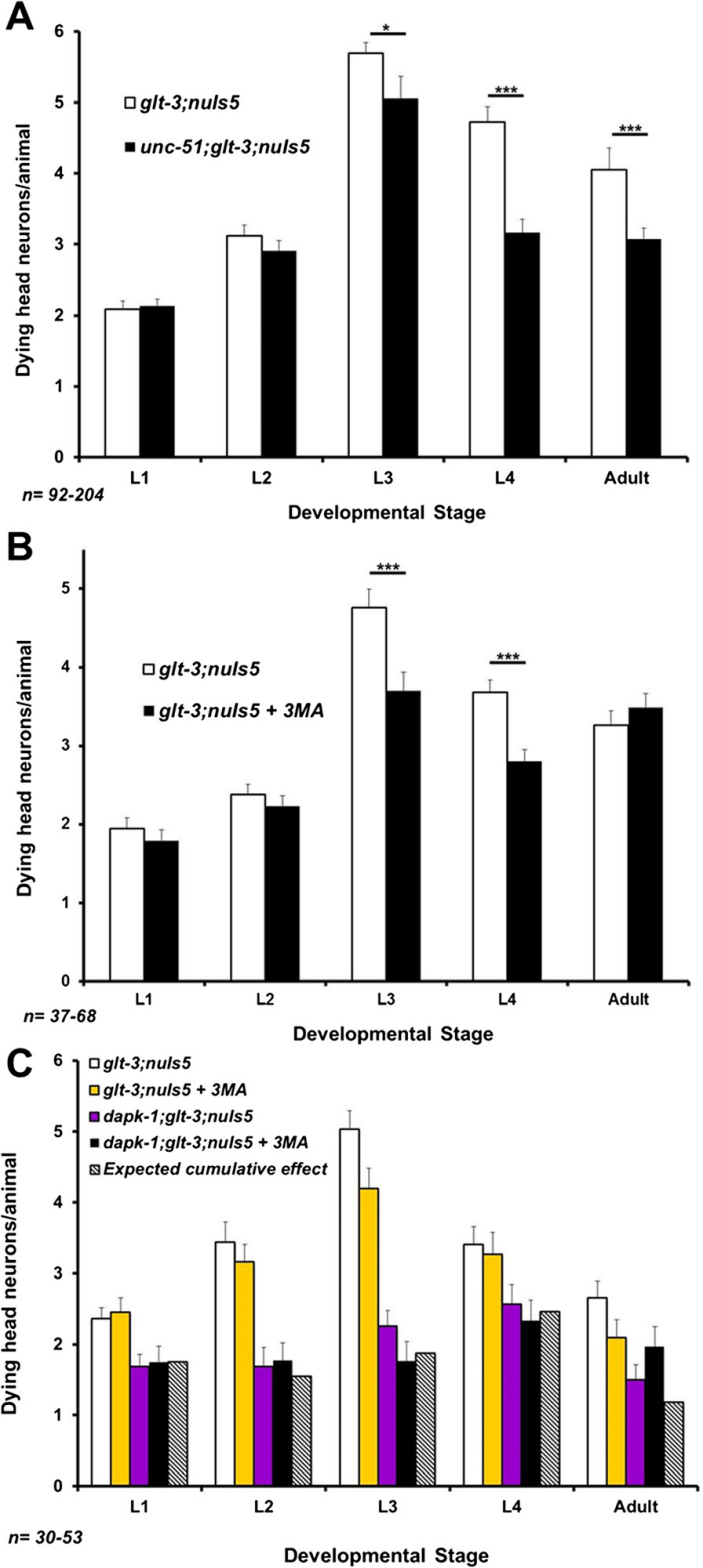
Treatments that block autophagy (and dramatically reduce neurodegeneration in other forms of necrotic neurodegeneration in *C. elegans*) have a reproducible but small effect in nematode excitotoxicity. **A)** A mutation in *unc-51* shows a moderate effect on nematode excitotoxicity. *p < 0.05 ; ***p < 0.01 **B)** Treatment with the autophagy-blocking drug 3MA has a moderate effect on nematode excitotoxicity. ***p < 0.01 **C)** Independent repetition of the experiment shown in B with the addition of epistasis analysis. The data shows that the only reproducible effect of autophagy blockade is in L3. Although at this stage the combined effect of autophagy blockade and *dapk-1* correlates with a model of independent action of these two factors, the moderate extent of effects limits the strength of such a conclusion.

To further study the possible involvement of autophagy in nematode excitotoxicity we examined the effect of inhibiting autophagy by genetic and chemical means. While a number of mutations and drugs have been used in the past, not all of them are available to us here. For example, *bec-1 ko* was previously used to monitor the requirement of this autophagy regulator for *mec-4(d)* –induced necrosis in early development [58]. However, the lethal effect of this mutation in later development, when most of the nematode excitotoxic necrosis occurs, prevents us from using this approach. Similarly, the vacuolar-type ATPase inhibitor bafilomycin is commonly used to block autophagy by elevating lysosomal pH [63]. However, the same V-ATPase is used in neurons to acidify synaptic vesicles as a means to provide the driving force for neurotransmitter loading [64-66], and therefore using bafilomycin can be expected to reduce neurotransmitter release. We therefore turned to use other means of intervention that are more compatible with our system: a mutation in the autophagy regulator *unc-51* (using the *e369* allele) and the chemical inhibitor 3MA (both used previously to show that degenerin-triggered neurodegeneration in *C. elegans* depends strongly on autophagy [58,60]). We noticed only a moderate effect for these two factors, evident in some developmental stages (Figure 4A and B). Such a moderate effect is in line with the reported effect of *unc-51* on *nuIs5* alone [60] (see discussion). We then used an independent set of experiments and epistasis analysis to determine if this moderate effect works independently of *dapk-1* or in the same pathway. We noticed that the effect of blocking autophagy on the extent of excitotoxicity is reproducible only in one developmental stage (L3). Trying to determine if *dapk-1* and 3MA work in the same pathway, we compared their observed combined effect (in the *dapk-1* + 3MA combination) to the calculated expected effect if these two processes were completely independent. However, given the moderate size of the 3MA effect and the inherent variability in our experiments, it is currently difficult to determine if *dapk-1* and autophagy work in the same pathway or independently. Nonetheless, the fact that the effect of autophagy is much more limited in size and duration than that of *dapk-1* supported a continued search for other mechanisms by which *dapk-1* might regulate excitotoxicity.

### The DAPK interaction-partner and phosphorylation-dependent peptidyl-prolyl isomerase Pin1/PINN-1 is a conserved suppressor of neurodegeneration

**Figure 5:**
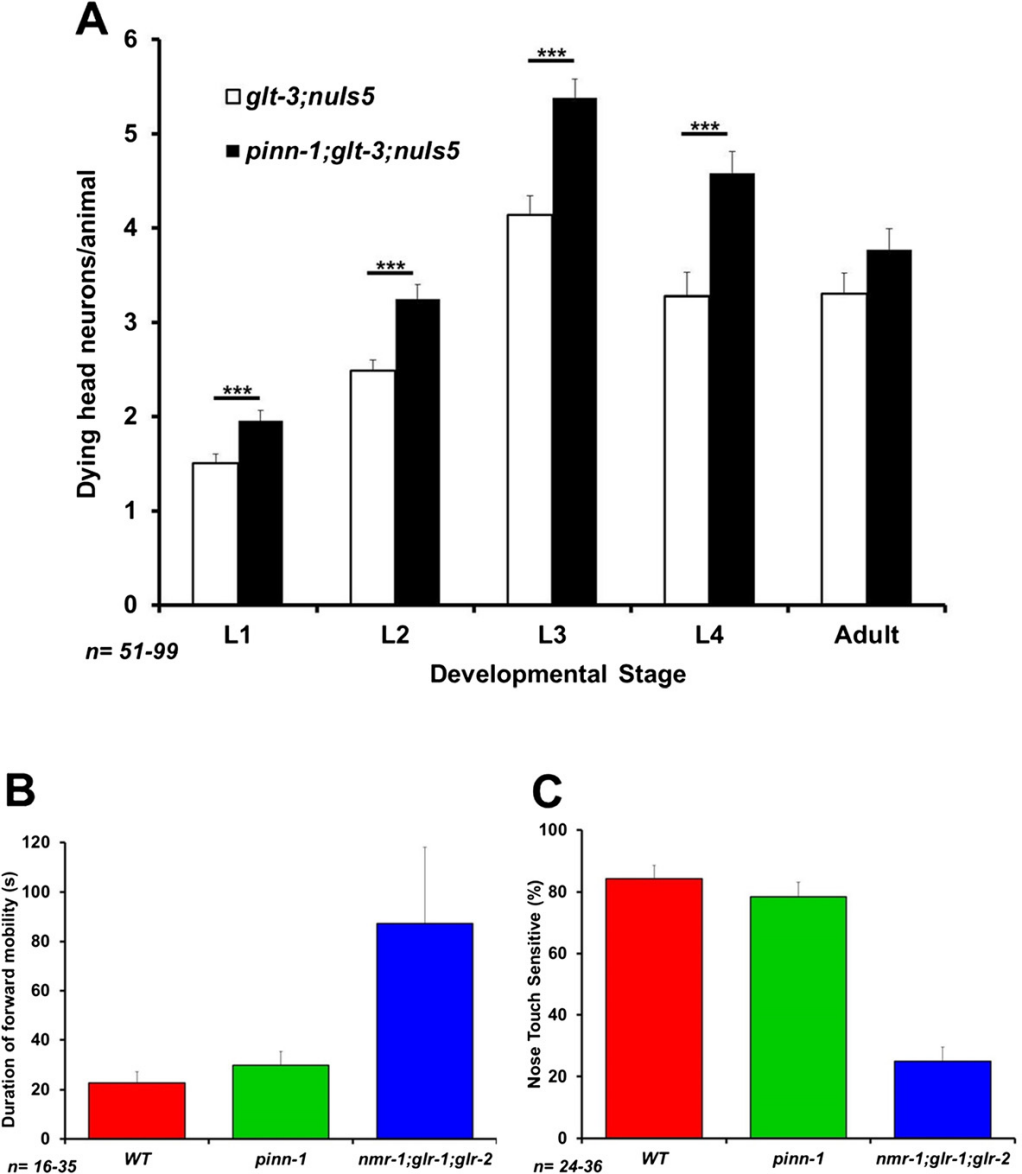
*pinn-1* is an important suppressor of nematode excitotoxicity that does not affect basic synaptic strength. **A)** *pinn-1* mutation enhances excitotoxicity throughout development. **p < 0.01 **B** & **C)** *pinn-1* does not affect the duration of spontaneous forward mobility or nose touch sensitivity, two sensitive measures of Glu synaptic strength.

Pin1 is an isomerase that changes the conformation of proline residues located next to phosphorylated Ser or Thr residues, thus changing overall protein conformation and controlling the activity of many phosphoproteins [67,68]. In recent years this protein has gained recognition as a major regulator of many signaling cascades, involved in both normal cell physiology, pathology, and in neurodegenerative diseases [67,69-71]. Pin1 is expressed in dendrites, its activity is modulated by Glu signaling, and it regulates PKCζ and PKMζ [72,73]. Pin1 is also known for its regulation of neuronal cytoskeleton and Tau protein phosphorylation, and for modulating neurodegeneration [69,74,75]. Recently, Pin1 was shown to functionally interact with DAPK [76]. We find that *pinn-1 ko* [77] causes increased neurodegeneration in nematode excitotoxicity (Figure 5). Like *dapk-1 ko*, the effect of *pinn-1 ko* is seen in all developmental stages (Figure 5A), and does not seem to involve a change in Glu synaptic strength (Figure 5B and C), suggesting that they influence cell-death processes subsequent to- (and not at the level of-) GluR.

**Figure 6:**
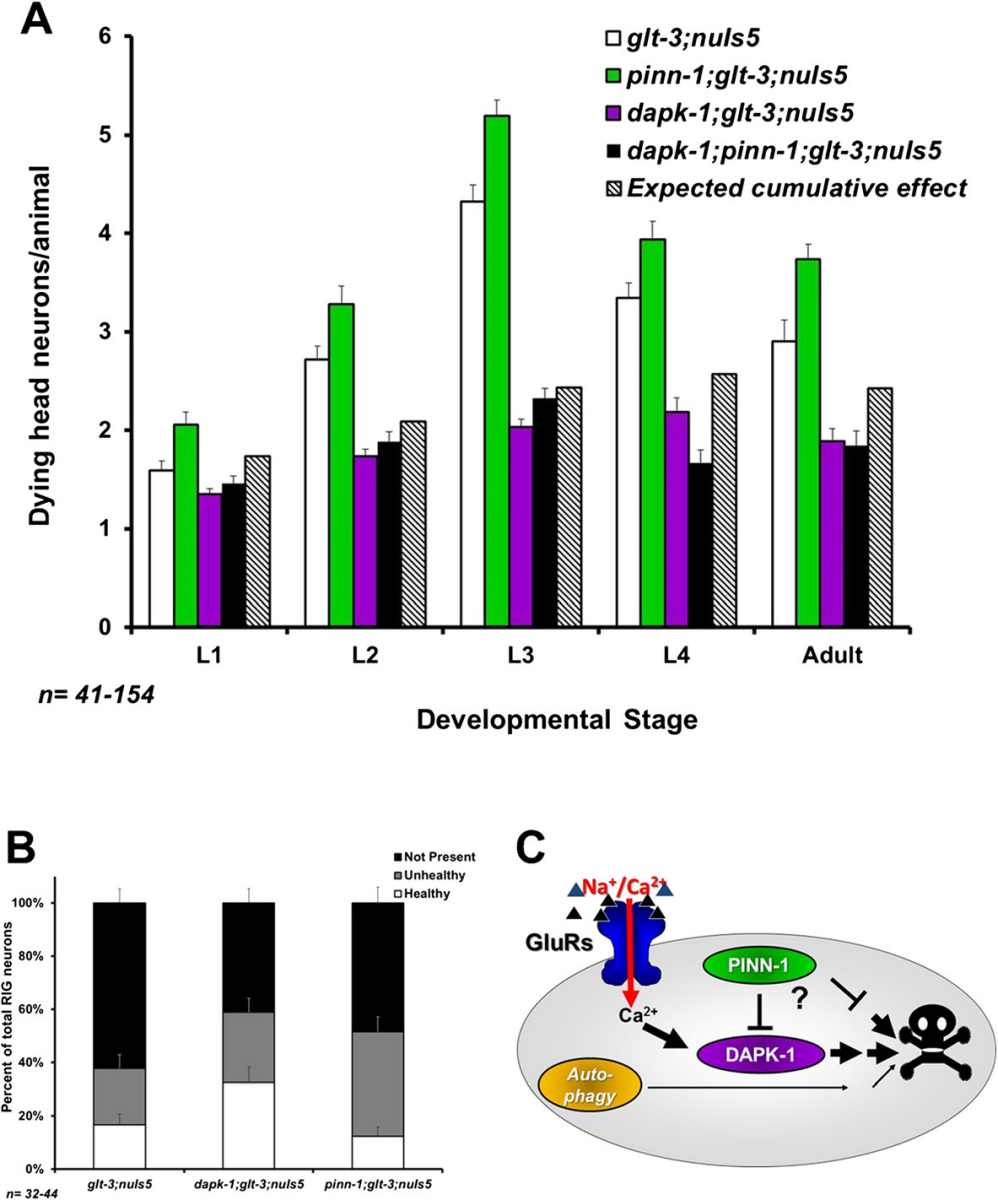
*dapk-1* might cooperate with *pinn-1* to regulate nematode excitotoxicity. **A)** Epistasis analysis testing the involvement of *dapk-1* and *pinn-1* in nematode excitotoxicity. Since the combined mutant shows strong suppression of neurodegeneration compared to the starting strain, these observations strongly suggest that *pinn-1* is not downstream of *dapk-1* (though the data is not conclusive enough to choose between the possibilities of *pinn-1* acting upstream or independently of *dapk-1*). **B)** Verification that the effects of *dapk-1* and *pinn-1* mutations on the dynamics of overall neurodegeneration in head neurons translates to survival of identified neurons in the adult (counting surviving GFP-labeled RIG neurons in young adult animals). **C)** One of the likely models that can account for our observations on the role of DAPK-1, PINN-1, and autophagy in nematode excitotoxicity.

We wished to confirm that the effects of *dapk-1* and *pinn-1* on the dynamics of vacuolar appearance (as seen in Figures 1A and 5A) translate to ultimate survival of specific neurons in adult animals. Given the stochastic nature of neurodegeneration in *glt-3;nuIs5* animals, it is usually difficult to identify which of the ~30 at-risk head neurons are degenerating in different animals. To circumvent this difficulty, we address the survival of specific neurons by focusing on the easily identifiable RIG neurons. The RIG neurons are part of the group of at-risk head neurons, they show only very minor levels of neurodegeneration in *nuIs5* alone, and they are sensitive to all the treatments that modify total head neuron degeneration analyzed in our previous studies [37,40,41]. Indeed, RIG neurons exhibit inverse correlation between the number of vacuole-like structures they show during development (as observed by Nomarski) and the number of GFP-labeled RIG neurons surviving in the adult [37]. We now confirm that the number of degenerating RIG neurons during development is reduced by *dapk-1 ko* and increased by *pinn-1 ko* (data not shown). Importantly, we find that the number of GFP-labeled RIG neurons that survive to adulthood is increased by *dapk-1 ko* and decreased by *pinn-1 ko* (Figure 6B). These observations suggest that the effects of *dapk-1* and *pinn-1* on vacuolar appearance during development indeed translate to changes in ultimate survival of identified neurons.

Finally, we wanted to determine if *dapk-1* (where a KO causes decreased neurodegeneration) and *pinn-1* (where a KO causes increased neurodegeneration) work in the same or separate pathways. Mammalian studies suggest that DAPK is acting upstream of Pin1 to inhibit its function [76], so that KO of *pinn-1* could be expected to exert its death-stimulating effect regardless of whether *dapk-1* is present or not. Instead, using epistasis analysis (Figure 6A), we observe that the double knockout *dapk-1* ; *pinn-1* exhibited suppression of excitotoxicity. We tried to further determine if the effect of *dapk-1*; *pinn-1* double knockout reflects an intermediate outcome (in line with an independent, parallel effect of these two factors) or a *dapk-1 ko* –only-like outcome (in line with an obligatory sequential effect, where the *dapk-1* mutation-induced decrease in neurodegeneration completely masks the ability of *pinn-1* mutation to increase neurodegeneration). To that end we calculated what would be the effect of these two factors acting in parallel, to predict their cumulative independent effect (Figure 6A). Unfortunately, the small difference between the observed effect of *dapk-1* alone and the calculated independent cumulative effects of *dapk-1* and *pinn-1*, together with the variability in our data, do not allow us to discriminate with confidence between these two options. We therefore limit our conclusion to say that *dapk-1* acts either downstream or in parallel to- (but not upstream of-) *pinn-1* in nematode excitotoxicity.

## Discussion

Mammalian studies have generated a plethora of proposed mechanisms in excitotoxicity, and some of these studies include suggested pathways for the involvement of DAPK in critical cell death events. However, the significance of these proposed mechanisms across divergent excitotoxic conditions remains unclear. In our study we focused on a glutamate-dependent neuronal death in *C. elegans* and examined a set of candidate mechanisms to define those that might be conserved through a large evolutionary distance. We previously found that some core constitutes of excitotoxicity are well conserved in our nematode model of GluT KO –triggered and CPAR-mediated neuronal necrosis. These include both death-promoting factors (such as release of Ca^2+^ from the ER [37]), and neuroprotective factors (such as cell stress resistance to insults, resistance that is conferred by FoxO/DAF-16 [40,41]). We now report that DAPK is also a highly conserved regulator of excitotoxicity (Figure 1A and B), though we note that *dapk-1 ko* does not bring neurodegeneration all the way down to background levels. We find no evidence for *dapk-1* –mediated regulation of synaptic strength, as defined by the spontaneous mobility assay (Figure 2B), suggesting that the effect of DAPK on mammalian NR2B/GluN2B, though probably very important, does not extend to all forms of excitotoxicity. We also found no role for the DANGER-related protein MAB-21 in nematode excitotoxicity (though DANGER is a much larger protein than MAB-21, suggesting it might have additional functions not tested here).

Although *dapk-1* is an important regulator of autophagy in *C. elegans*, we find only a minor role for autophagy in the neurodegenerative condition we study in *glt-3;nuIs5* animals. We find that blocking autophagy has a relatively small neurodegeneration-reducing effect (~0.5-1 dying neuron/animal, Figure 4). This effect is smaller than the neurodegeneration-suppressing effect of *dapk-1 ko* (a decrease of ~2.5 dying neurons/animal, three independent isolates counted in Figures 1A, 4C, and 6A). Therefore, even if related, autophagy cannot account for the majority of DAPK’s effect in *glt-3;nuIs5* animals. The minor effect of autophagy on neurodegeneration in *glt-3;nuIs5* is in sharp contrast to the major effect that autophagy has on degenerin-mediated neurodegeneration, where it suppresses neurodegeneration by 75-90% [60]. We further note that these previous studies have demonstrated that blocking autophagy decreases the extent of low-level neurodegeneration triggered by *nuIs5* alone [60]. Since neurodegeneration by *nuIs5* alone (independent of excitotoxicity) is part of the total number of degenerating neurons we count in our assay (accounting for ~1 dying neuron/animal [37]), the small effect of autophagy seen in the current study can be attributed to its documented effect of *nuIs5* alone. Indeed, we see no significant difference in labeling of fluorescent autophagy reporter when comparing *nuIs5* to *glt-3;nuIs5* animals (Figure 3). The results of epistasis analysis are not conclusive, but put together with the difference in size and timing of the effects of *dapk-1* and autophagy on nematode excitotoxicity, our observations cause us to favor one of the possible models in which autophagy has only a minor role in nematode excitotoxicity in parallel to the role of DAPK-1 (Figure 6C). We emphasize that the parallel action of DAPK and autophagy is preferred by us in the specific scenario of nematode excitotoxic stress, while other stresses might include DAPK and autophagy in other pathway configurations.

The observation that the DAPK-interaction-partner Pin1/PINN-1 is a significant regulator of excitotoxicity is particularly intriguing and novel. In addition to its involvement in cancer [71] and stress response [78], Pin1 has been shown to be a critical regulator of dendritic Glu responses [72,73] and of neurodegeneration in Alzheimer disease [69,74,75]. The results of our epistasis analysis do not seem to support a role for Pin1/PINN-1 as an obligatory step downstream of DAPK/DAPK-1, as might be inferred from mammalian cancer studies [76]. Instead, our data supports that PINN-1 acts in parallel or upstream of DAPK-1 (Figure 6A and C).

## Conclusions

Put together, our study suggests that nematode excitotoxicity can be an important tool to sift through many proposed mechanisms of excitotoxicity, allowing us to identify conserved mechanisms that might be at the core of neurodegenerative processes common across divergent excitotoxic scenarios. Furthermore, our studies now illuminate DAPK/DAPK-1 and Pin1/PINN-1 as two such factors, important for excitotoxicity mechanisms across a large evolutionary distance. We can now further use this system to decipher DAPK-1 and PINN-1 –related mechanisms in nematode excitotoxicity, with the hope that such understanding might help us to continue uncovering conserved core mechanisms in this important form of neurodegeneration.

## Methods

### Strains

The following *C. elegans* strains were obtained from the *C. elegans* Genetic Center (CGC), from the Japanese National Bioresource Project (NBSP), or from the original producers: **WT**: Bristol N2 (RRID:CGC_N2 (ancestral)); **Nematode excitotoxicity model:** ZB1102: *glt-3(bz34) IV; nuIs5 V;* (RRID:CGC_ZB1102) ***dapk-1****:* VC432: *dapk-1(gk219) I;* (RRID:CGC_VC432) ***dapk-1 over-expression:*** CZ9277**:***frIs7*[P_*nlp-29*_*::GFP*; P_*col-12*_*:DsRed*] (*IV)*; *juEx1933*[P_*hsp16*_*::DAPK-1*; P_*ttx-3*_*::RFP*] (the *frIs7* insertion was later eliminated during our cross); **Aldicarb assay control strains:** sv-hyper-releasing/aldicarb-oversensitive RB1367: *cpx-1(ok1552)* (RRID:CGC_RB1367)**;** sv-under-releasing/aldicarb-resistant NM791: *rab-3(js49)* (RRID:CGC_NM791): **Autophagy modulator*unc-51***: CB369: *unc-51(e369) V* (RRID:CGC_CB369); **Autophagy label, DsRed::LGG-1:** (originally created by the Tavernarakis lab [62], strain naming here as per BMC policy) IMN21: N2; *Ex [P*_*lgg-1*_*:: DsRed:: LGG-1; P*_*myo-2*_*:: GFP]*; **Glu-regulated mobility defective control:** VM1268: *nmr-1(ak4) II; glr-2(ak10) glr-1(ky176) III;***DANGER-related protein MAB-21:** EM128: *mab-21(bx53) III* (Q203* , CAG -- > UAG) (RRID:CGC_EM128)*.****CaMKK-like KO***: *ckk-1(ok1033) III* (RRID:CGC_VC691)*;****Pin1-like KO:****pinn-1(tm2235)* II (this 364 bp deletion runs the ORF into a stop codon after 8 codons). Most crosses were followed by PCR analysis to detect deletions, and by monitoring *nuIs5’s* GFP expression in *glr-1*-expressing interneurons using a high power fluorescence dissecting scope. *dapk-1* over-expressing construct *juEx1933* was followed by its coinjection marker, which produces RFP expression on AIY neurons. The *unc-51* mutation was followed by uncoordinated phenotype and sequencing. DsRed::LGG-1 was followed by DsRed expression in cells throughout the body and by GFP expression in the pharynx (from the *myo-2* promoter). We constructed the following strains: ***dapk-1 in excitotoxicity*** (by crossing VC432 and ZB1102) IMN26: *dapk-1(gk219) I; glt-3(bz34) IV; nuIs5 V;****DAPK-1 overexpression in excitotoxicity*** (by crossing ZB1102 and CZ9277) IMN25: *glt-3(bz34) IV; nuIs5 V*; *juEx1933* [P_*hsp16*_*::DAPK-1*; P_*ttx-3*_*::RFP*]; ***Autophagy suppression in excitotoxicity*** (by crossing ZB1102 and CB369) IMN22: *glt-3(bz34) IV; nuIs5 , unc-51(e369) V;***Autophagy reporter in excitotoxicity** IMN24: *glt-3(bz34) IV; nuIs5 V; Ex[P*_*lgg-1*_*:: DsRed:: LGG-1; P*_*myo-2*_*:: GFP];****CaMKK deletion in excitotoxicity:****ckk-1 (ok1033) III; glt-3(bz34) IV; nuIs5 V.****DANGER-like deletion in excitotoxicity:*** (by crossing EM128 and ZB1102): *mab-21(bx53) III; glt-3(bz34) IV; nuIs5 V*; ***Pin1-like deletion in excitotoxicity:*** IMN30: *pinn-1(tm2235) II; glt-3(bz34) IV ; nuIs5 V* (± *dapk-1(gk219) I*).

### Neurodegeneration analysis

The level of necrotic neurodegeneration in head neurons was monitored using an inverted scope and Nomarski Differential Interference Contrast (DIC). Animals with no anesthetics were examined on a fresh chunk of agar of nematode culture flipped upside down over a slide. Swollen “vacuolated-looking” cells located in the area of the nerve ring were counted as head neurons undergoing necrosis, as described previously [37,41]. Briefly, we use freshly growing mixed-stage animals, and we record the developmental stage and the number of “vacuolated” dying neurons of each animal that we see while scanning through the agar chunk, thus creating a “snap-shot” of the transitory number of dying neurons exhibited by the population of animals at the time of analysis. As mentioned above, in the excitotoxicity strain the number of “vacuolated” cells observed at each developmental stage typically goes up until L3 (with the maturation of Glu signaling in the worm), and then declines with the engulfment of cell copses. This analysis is repeated several times, with several isolates of each strain. For confirmation of suspected effects in new mutant combinations, a representative part of the analysis is done blindly by independent observers. Similar numbers of control and test animals are recorded in each session. Records from all these sessions is pooled together to calculate average neurodegeneration from large number of animals in each strain and each stage (50–200 in each data bar). We occasionally confirmed these dying neurons as neurons postsynaptic to Glu connections by verifying their labeling with the GFP co-expressed in *nuIs5* animals under the *glr-1* promoter. For heat-shock-induced overexpression of DAPK-1, animals carrying [P_*hsp16*_*::DAPK-1*; P_*ttx-3*_*::RFP*] were placed in a 35°C incubator for 2 hours to induce activation of heat shock promoter, left to rest for about 30 minutes and scored regularly for two days for the extent of swollen degenerating neurons (protocol & strains coordinated with the Chisholm group, UCSD [42]). Experimental animals (animals with extrachromosomal array) and control animals (animals lacking extrachromosomal array) were obtained, identified and scored on the same day from the same pool of heat-shocked animals. For epistasis analysis between given excitotoxicity-modifying mutations X and Y, we calculated the effect of each mutation for each developmental stage as: Fold effect of X = (the average number of dying head neuron in the excitotoxicity strain in the presence of mutation X)/(the average number of dying head neuron in the starting excitotoxicity strain), with a similar calculation for mutation Y. The expected cumulative fold effect of mutations X and Y when working independently in parallel pathways is (fold effect of X) * (fold effect of Y). The calculated expected number of dying neurons presented on the graphs in Figures 4 and 6 is (the average number of dying head neuron in the starting excitotoxicity strain) * (fold effect of X) * (fold effect of Y). Error bars represent SE. Statistical significance of difference between control groups and experimental groups was analyzed using *z-test* score (as the significance of these differences is calculated for large populations of ~100 animals in each group).

### Fluorescence microscopy analysis of autophagy

Animals carrying *Ex [P*_*lgg-1*_*:: DsRed:: LGG-1; P*_*myo-2*_*:: GFP]; glt-3(bz34) IV; nuIs5 V and Ex [P*_*lgg-1*_*:: DsRed:: LGG-1; P*_*myo-2*_*:: GFP]; nuIs5 V* were analyzed for the extent to which *glr-1* expressing command interneuron (labeled with GFP) were undergoing autophagy (i.e., show DsRed punca or increased DsRed intensity). Animals were mounted on a 2% agar pad. To compare average DsRed intensity between worms we identified the cell with the highest DsRed intensity as 100% and then compared the other neurons to this cell (following the procedure used by the Tavernarakis lab, who developed this DsRed::LGG-1 marker [62]). A few cells were analyzed in each animal and the average intensity from a total from 20–25 animals was calculated. We also counted the average of the number of green-labeled neurons showing DsRed puncta as another indication of neurons undergoing autophagy and compared control (*nuIs5* only) versus experimental (*glt-3; nuIs5*) groups.

### Blockade of autophagy with 3MA

For 3-methyladenine (3MA) treatment, mixed stage animals from the excitotoxicity strain (*glt-3; nuIs5*) or the DAPK-inhibited excitotoxicity strain (*dapk-1; glt-3; nuIs5*) were incubated overnight with 10 mM 3-MA (Sigma, M9281) dissolved in 1% DMSO (experimental group) or 1% DMSO alone (control). Plates were supplemented with *E. coli* OP50. The extent of neurodegeneration was recorded 20–24 h after treatment.

### Behavioral assays

Behavioral assays on nematode locomotion and worm paralysis were performed blindly following standard methods of behavioral analysis. For duration of Glu-regulated spontaneous forward mobility we followed the protocol of the Maricq group (U Utah) [79]. We did not measure mobility in strains that show excitotoxic neurodegeneration because the degeneration or rescue of command interneurons can change the results of this assay. For aldicarb assays we followed the Nonet lab protocol (Washington U) [48]. Briefly, we soaked worm plates with aldicarb to a final concentration of 0.5 mM and added food. We placed ~30 freshly growing young adult animal onto these plates. The percentages of paralyzed worms were recorded every 15 minutes for a period of 2 hours.
